# Supplementary material for: Where octagonal geometry meets chaos: A new S-Box for advanced cryptographic systems
Source: PLoS One. 2025 Jun 9;20(6):e0320457. doi: 10.1371/journal.pone.0320457 (PMC12148150; doi:10.1371/journal.pone.0320457)
Supplement: Attachment — Submitted filename: Response to Reviewers.pdf [file pone.0320457.s001.pdf]

Reviewer #1:

In simulation and performance analysis, authors are required to add fixed points, conduct reverse fixed-point analysis for further improvements, and check validity against various cryptographic attacks.

Answer: Thank you for your esteemed review. Fixed points and reverse fixed-point analyses have been carried out (Kindly see the subsection 4.7). Besides, cryptographic attacks analyses have also been made (Kindly see the subsection 4.8).

Reviewer #2:

The paper has a new design of S-Box based on chaos algorithm and Octagonal Geometry. The authors discuss the design and analyze the results. I think that there is a scientific contribution to be publish after correcting the following:

1. Overall text need to justify.

Answer: Thank you for your esteemed review. We have used the Latex template of the journal. So, this is the default behavior of the journal.

2. Section 5 (Discussion), I think this section may be merge with the section 4 (Optional).

Answer: Thank you for your valuable suggestion regarding merging Section 5 (Discussion) with Section 4 (Analysis). However, we believe that keeping these sections separate enhances the clarity and structure of the article. Section 4 focuses on the detailed technical analysis and evaluation of the proposed method, while Section 5 provides a broader interpretation of the results, their implications, and their relevance to the field. This separation ensures that both aspects are thoroughly addressed without overlap, offering a more comprehensive and organized presentation of the work.

3. Check all abbreviations.

Answer: Concern has been addressed.

4. The paper should be submitted carefully to the template of the journal.

Answer: Concern has been addressed.
